# Integrin α2β1 in nonactivated conformation can induce focal adhesion kinase signaling

**DOI:** 10.1038/s41598-017-03640-w

**Published:** 2017-06-13

**Authors:** Maria Salmela, Johanna Jokinen, Silja Tiitta, Pekka Rappu, R. Holland Cheng, Jyrki Heino

**Affiliations:** 1 Department of Biochemistry, University of Turku, Turku, Finland; 2 Department of Molecular and Cellular Biology, University of California, Davis, CA USA

## Abstract

Conformational activation of integrins is generally required for ligand binding and cellular signalling. However, we have previously reported that the nonactivated conformation of α2β1 integrin can also bind to large ligands, such as human echovirus 1. In this study, we show that the interaction between the nonactivated integrin and a ligand resulted in the activation of focal adhesion kinase (FAK) in a protein kinase C dependent manner. A loss-of-function mutation, α2E336A, in the α2-integrin did not prevent the activation of FAK, nor did EDTA-mediated inactivation of the integrin. Full FAK activation was observed, since phosphorylation was not only confirmed in residue Y397, but also in residues Y576/7. Furthermore, initiation of downstream signaling by paxillin phosphorylation in residue Y118 was evident, even though this activation was transient by nature, probably due to the lack of talin involvement in FAK activation and the absence of vinculin in the adhesion complexes formed by the nonactivated integrins. Altogether these results indicate that the nonactivated integrins can induce cellular signaling, but the outcome of the signaling differs from conventional integrin signaling.

## Introduction

The well-established model of integrin function stresses the importance of the conformational change from a nonactivated, bent conformation to an active, extended state prior to ligand binding and outside-in signaling^1^. Despite the fact that the conformational activation of integrins is often required for ligand binding, nonactivated integrins may have specific biological functions as well. Recently, matrix fibrillogenesis was shown to be suppressed by inactive α5β1 integrins^2^, and in addition inactive αvβ3 has been reported to interact with cell-surface protein Thy-1^3^. Furthermore, our previous data suggested that under shear stress, α2β1 integrin binds to collagen I without the conformational preactivation of the receptor^4^. Additionally, ligand-independent signaling mechanisms have recently been found. They can be activated by the interaction of integrins with other transmembrane proteins, such as urokinase-type plasminogen activator receptor^5^.

According to the conventional model of integrin outside-in signaling, the conformational changes in the ligand binding inserted domain (αI domain, often called as αA domain), and consequently in the βI domain, lead to the separation of integrin legs, including transmembrane and intracellular domains. This allows intracellular signaling molecules to interact with integrins, which then initiates the formation of focal adhesion sites^6^. One of the first signaling events is the autophosphorylation of tyrosine residues 397 in focal adhesion kinase (FAK)^7^. The molecular mechanisms leading to the activation of FAK are complex and have yet remained in many parts unresolved^8, 9^. It has recently been shown that FAK activation requires its recruitment to the cell membrane, conformational changes in FAK to discontinue the autoinhibition, and the dimerization of two FAK molecules^10–12^. Following the phosphorylation of Y397, interaction with Src induces the phosphorylation of additional tyrosine residues, Y576 and Y577, leading to full activation of FAK and to the further FAK interaction with other signaling molecules^13, 14^.

We have previously showed that human echovirus 1 (EV1) binds with a higher avidity to the closed than to the open αI domain in the α2β1 integrin. Similarly, EV1 favours the nonactivated, presumably bent α2β1 over the preactivated integrin. Moreover, EV1 binding does not seem to cause a conformational change in α2β1, whereas one virus particle can cluster several integrins together^15^. This gave us an opportunity to utilize the interaction between EV1 and α2β1 integrin to analyse the signaling mechanisms related to nonactivated integrins.

In this study we show that the ligand binding and clustering of the nonactivated α2β1 integrins can activate FAK and paxillin. We report that the adhesion site generated by the nonactivated α2β1 integrins is different when compared to the conventional focal adhesions mediated by integrin binding to the extracellular matrix. FAK activation by the nonactivated integrins is talin-1 independent and it can be affected by PKC inhibitors. Furthermore, the phosphorylation of paxillin seems to be transient in nature, unlike after cell adhesion to collagen I.

## Results

### The phosphorylation of FAK is initiated by the nonactivated α2β1 integrin

**Figure 1:**
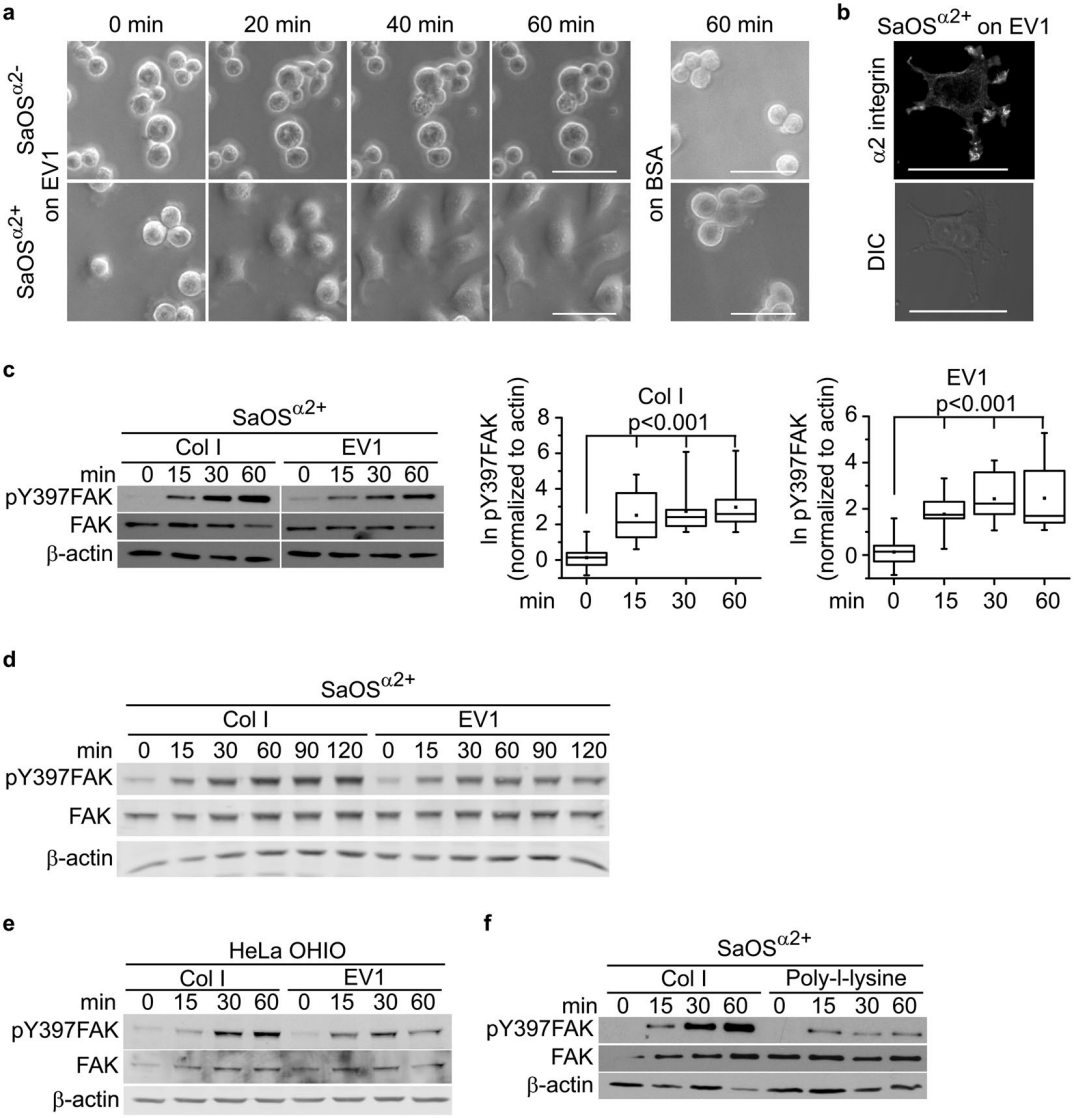
Cells expressing α2 integrin attach on EV1 coated surface through interaction between nonactivated integrins and the virus, and activate FAK signaling. (**a**) Integrin α2 transfected SaOS cells (SaOS^α2+^) and α2 integrin negative wild type SaOS cells (SaOS^α2−^) spreading on EV1 or BSA coated surface. Photographed through a phase contrast microscope after 0, 20, 40, and 60 min. Scale bar 50 μm. (**b**) Confocal microscopy image of SaOS^α2+^ cell on EV1-coated surface. α2 integrins were labelled using specific antibodies. Scale bar 50 μm. (**c**) Western blotting detection of phosphorylation of FAK at Y397 in SaOS^α2+^ cells on collagen I or an immobilized layer of EV1. Typical experiment on the left, and on the right, a visualization of the results from ten independent experiments presented in box blot as natural logarithm of pY397FAK band intensity normalized to β-actin. Whiskers present minimum and maximum of all of the data. Non-parametric Mann-Whitney U test was used to determine statistical significance. (**d**) Western blotting analysis of FAK Y397 phosphorylation SaOS^α2+^ cells plated on collagen I or an immobilized layer of EV1, time points 0 to 120 min. (**e**) Western blotting of FAK Y397 phosphorylation in HeLa OHIO cells plated on collagen I or an immobilized layer of EV1. (**f**) Western blotting of FAK Y397 phosphorylation in SaOS^α2+^ cells spreading on Collagen I or poly-L-lysine.

To study the interaction between cell and EV1, SaOS cells were plated on a layer of immobilized EV1 particles. This experimental design was selected, instead of the use of cell monolayers exposed to virus particles, because it allowed us to study cells without the disturbance from other concomitantly occurring integrin mediated matrix adhesion mechanisms. We have previously shown, that Chinese Hamster Ovary cells overexpressing the α2 integrin can attach to immobilized layer of EV1^15^. Here, the α2 integrin transfected SaOS cells (SaOS^α2+^) were able to spread on EV1, unlike the α2β1 negative wild type SaOS cells (SaOS^α2−^) (Fig. 1a). No SaOS cells attached on the plates coated with 0.1% BSA (Fig. 1a). The results are in accordance with the previous observations indicating that α2β1 is the only cellular receptor for EV1^16, 17^. Based on the confocal microscopy and immunofluorescence utilizing specific antibodies, the α2β1 integrin is located at the tips of the cellular protrusions, suggesting that these are the main plasma membrane areas involved in the EV1 interaction with the cell (Fig. 1b). Integrin β1 subunit also colocalized with α2 subunit (Fig. S1a). Cells spreading on an EV1 layer did not form clear focal adhesions, or actin stress fibers. Rather, actin formed thin, unorganized structures (Fig. S1b). EV1 was further covalently crosslinked on a glass surface to reduce the softness of the matrix. Even in the more rigid environment, the nonactivated integrins could not induce the formation of the focal adhesions or the actin stress fibers (Fig. S1c). Smaller physical forces mediated by cell-ECM adhesions and modulating the intracellular molecular interactions could explain the observation.

Western blot analysis of the SaOS^α2+^ cells plated on an EV1 layer showed that the tyrosine residue Y397 in FAK is phosphorylated in less than 15 min and that the phosphorylation level increases up to 60 min. The activation was statistically significant. Up to 60 min, the time schedule and magnitude of FAK phosphorylation was similar to SaOS^α2+^ cells spreading on a collagen I (Figs 1c, S2). At later time points (up to 120 min), FAK activation on EV1 seemed to decline, whereas on collagen I the activation stayed high (Fig. 1d). We also tested the phenomenon in another cell line, the HeLa Ohio that expresses endogenous α2 integrin and can also act as EV1 host cell. FAK activation could be detected when these cells were plated on an EV1 coated surface (Fig. 1e). We also verified that the activation of FAK is dependent on the receptor-mediated adhesion. In accordance with the previously published reports^18^, the levels of phosphorylated FAK were much lower in the cells plated on a poly-L-lysine (Fig. 1f) than in cells plated on a collagen I or an EV1 coated surface (Fig. 1c), and did not gradually increase. Cell adhesion to poly-l-lysine is integrin independent and does not induce integrin dependent signaling.

### The phosphorylation of FAK is initiated in the conditions that are known to constrain integrin to the bent conformation

**Figure 2:**
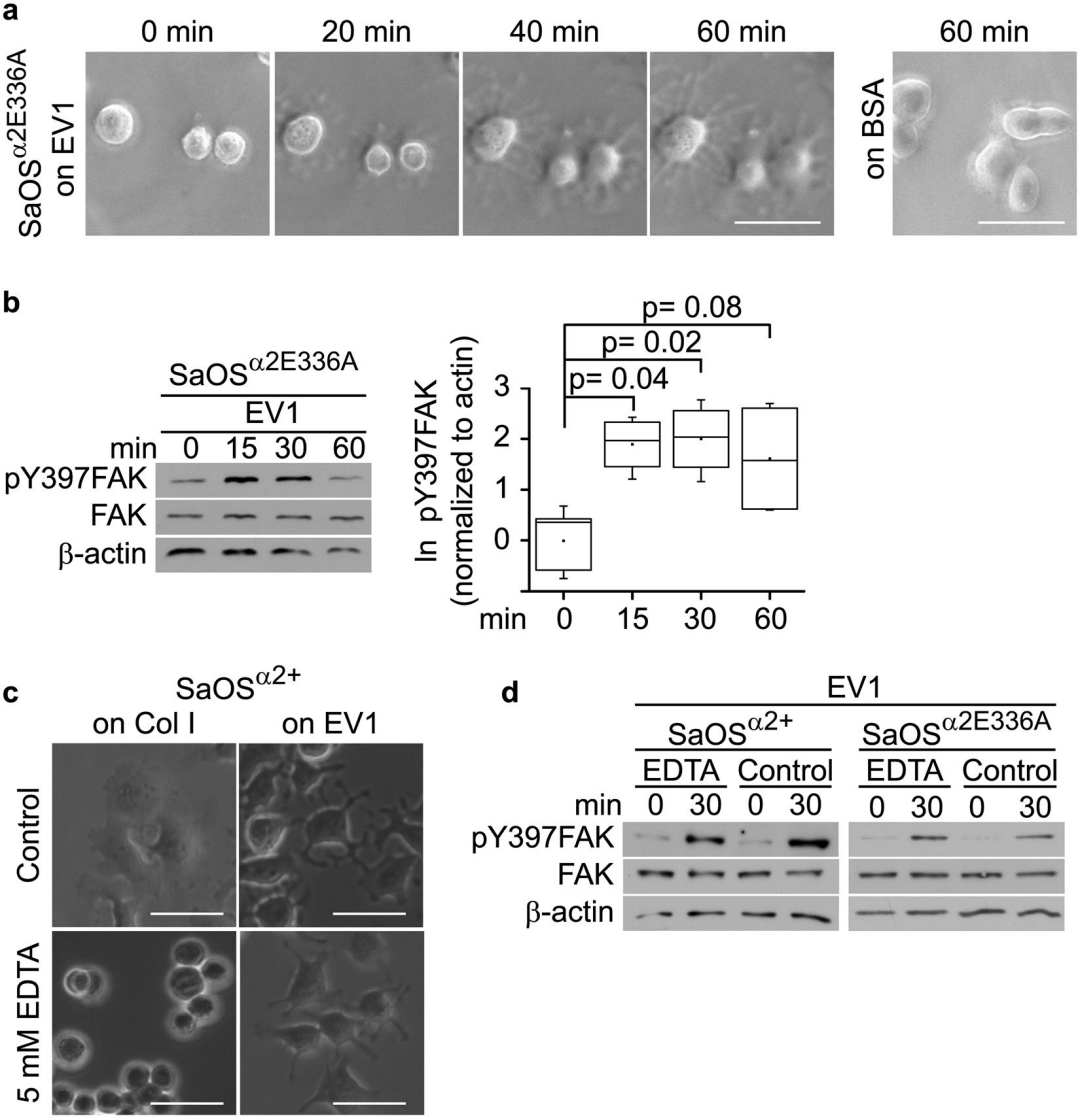
FAK is activated through α2 integrins harboring the loss-of-function mutation E366A in the α2 subunit, and in the presence of EDTA. (**a**) SaOS cells with the loss-of-function mutation E336A in the α2 subunit (SaOS^α^ ^2E336A^) on EV1-coated surface. Photographed through a phase contrast microscope after 0, 20, 40, and 60 min. Scale bar 50 μm. (**b**) Western blot analysis of pY397FAK in SaOS^α^ ^2E336A^ cells plated on EV1. Typical experiment on the left, and on the right, a visualization of the results from four independent experiments presented in box blot as natural logarithm of pY397FAK band intensity normalized to β-actin. Whiskers present minimum and maximum of all of the data. Non-parametric Mann-Whitney U test was used to determine statistical significance. (**c**) Phase contrast microscopy images of SaOS^α^ ^2+^ cells treated with integrin inactivating EDTA (5 mM) after spreading for 60 min on an immobilized layer of EV1 or collagen I. Scale bar 50 μm. (**d**) Western blot analysis of pY397FAK in SaOS^α^ ^2+^ and SaOS^α^ ^2E336A^ cells treated with 5 mM EDTA and plated on an immobilized layer of EV1.

To confirm that the α2β1 integrins initiating the activation of FAK are in the nonactivated conformation, we first introduced the mutation E336A into the α2 subunit and created a SaOS cell line overexpressing this variant integrin (SaOS^α2E336A^)^15^. Based on the previous observations, the corresponding mutations in αL and αM integrins shifts the balance in integrin conformation towards the bent state^19, 20^. We have earlier reported that in α2 subunit, E336A is a loss-of-function mutation which prevents the conformational activation of the α2β1 integrin^21^. We confirmed by the flow cytometric analysis equal α2 integrin expression levels in the SaOS^α2+^ and SaOS^α2E336A^ cells (Fig. S3a). We also confirmed that overexpressed α2 pairs with β1 integrins in SaOS cells (Fig. S3b). The SaOS^α2E336A^ cells (Fig. 2a) spread on an EV1 coated surface similarly to the SaOS^α2+^ cells (Fig. 1a). Cells did not spread on surfaces coated with BSA (Fig. 2a). Importantly, SaOS^α2E336A^ adhesion to EV1 activated the Y397 phosphorylation in FAK (Fig. 2b). Moreover, in conditions where the nonactivated conformation of the integrin was further induced by the loss-of-function mutation, the activation of FAK was clearly transient and declined after 30 min. The phosphorylation of FAK by the nonactivated integrins was further confirmed by treating the cells with 5 mM EDTA prior to plating them on an EV1 layer or a collagen I coated surface. The extended conformation of the integrin α-subunit leg is considered to be dependent on the presence of Ca^2+^ ^22^ and therefore EDTA can keep the integrins in the bent state. Here, SaOS^α2+^ cells treated with EDTA could attach and spread on an EV1 layer but not on collagen I (Fig. 2c). Accordingly, a previous study showed that the EDTA treatment does not prevent the adhesion of EV1 particles to the cell surface^23^. Both SaOS^α2+^ and SaOS^α2E336A^ cells showed FAK activation on an EV1 coated cell culture plate in the presence of 5 mM EDTA (Fig. 2d). Cells were not tested on a collagen I, since they did not adhere to collagen in the presence of EDTA. Thus, we conclude that the loss-of-function mutation of integrins or the inhibition of integrins with EDTA, both known to promote the bent conformation, do not prevent the ability of integrins to activate FAK.

### The nonactivated α2β1 integrins initiate the formation of the atypical adhesion sites in which FAK activation is talin-1 independent, but requires the PKC activity

To test whether the activation of FAK by the nonactivated integrins is dependent on the cytoplasmic domain of the α2 subunit, we swapped the intracellular tail from α2 to α1 and expressed the variant integrin in the SaOS cells^24^. Equal α2 integrin expression levels in the SaOS^α2+^ and SaOS^α2/α1tail^ cells were confirmed by flow cytometric analysis (Fig. S4a). We did not observe any changes in FAK activation after swapping the integrin tails (Fig. S4b). The short intracellular tails of the integrin α subunits have not been reported to directly bind to FAK, whereas previous studies have showed that FAK can directly bind to the integrin β tails^25^. Thus, in our model system it is probable that the intracellular interactions of the integrin β subunits are responsible for the phosphorylation of FAK.

**Figure 3:**
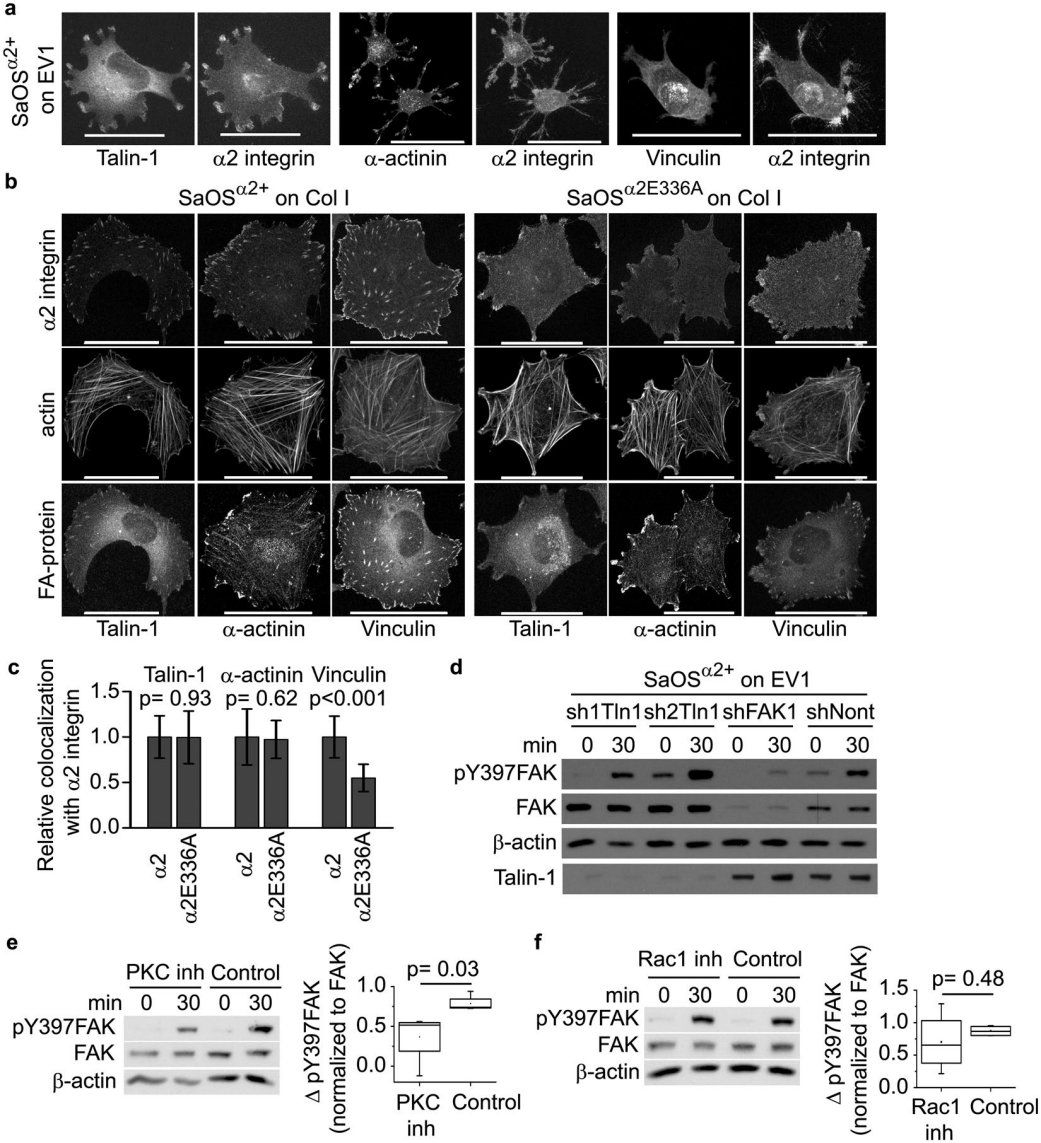
Protein interaction with nonactivated α2 integrins. (**a**) Confocal microscopy images of SaOS^α^ ^2+^ cells plated on EV1 coated cell culture plate showing talin-1, α-actinin, vinculin and α2-integrin localization. Scale bar 50 μm. (**b**) Confocal microscopy images of SaOS^α^ ^2+^ and SaOS^α^ ^2E336A^ cells plated on collagen I showing talin-1, α-actinin, vinculin and α2-integrin localization. Scale bar 50 μm. (**c**) Measurement of the localization of talin 1, α-actinin and vinculin to α2 integrin positive areas in confocal microscopy images similar to images shown in B (10–20 images from three independent experiments, analysed with BioImageXD). Statistical significance was calculated with unpaired Student’s t-test. (**d**) Western blotting of Y397 phosphorylation of FAK in talin 1 silenced SaOS^α^ ^2+^ cells spreading on an immobilised layer of EV1. Talin 1 was silenced with two different shRNAs, and as a control, one nontarget shRNA and one shRNA able to silence FAK were used. (**e,f**) Western blotting of Y397 phosphorylation of FAK in SaOS^α^ ^2+^ cells spreading on EV1 after treatment with PKC inhibitor safingol (**e**; 10 μM, 30 min, n = 4) or Rac inhibitor NSC23766 (**f**; 100 μM, 1 h, n = 4). Data are presented as box blot where whiskers present minimum and maximum values. The p value from paired one-tailed Student’s t-test is shown.

We used confocal microscopy to analyse whether talin-1 or other focal adhesion proteins can be detected in the cellular protrusions mediating the adhesion to EV1. Immunostaining with the specific antibodies indicated that talin-1 is present in the same areas at the cell protrusions where α2 integrin is found (Fig. 3a), but failed to show densely packed focal adhesion like structures. We also immunostained other selected focal adhesion proteins, α-actinin and vinculin, and visualized their colocalization with the nonactivated α2 integrins. In the EV1-induced cellular protrusions, α-actinin colocalized with α2 similarly to talin-1, but we could not see the colocalization of α2β1 integrin with vinculin (Fig. 3a). In addition to playing a critical role in the formation of focal adhesion plaques and in integrin outside-in signaling, talin also activates integrins^26^. Therefore, the presence of talin-1 in the adhesion sites formed by the nonactivated integrins bound to EV1, was an unexpected observation. The lack of vinculin, however, indicates that the talin conformation does not support the interaction with vinculin. Thus, talin is not stretched in a manner that would unveil its cryptic vinculin binding sites^27^. This could explain the lack of conventional focal adhesions and actin stress fibers on an EV1 layer (Fig. S1b,c).

To study further the putative interaction between talin and the nonactivated α2β1 integrin, we analysed SaOS^α^ ^2+^ and SaOS^α^ ^2E336A^ cells plated on collagen I. Wild type SaOS cells are negative for the α2 integrin, but they express other collagen receptor integrins, e.g. α1β1 and α11β1 that can mediate cell binding to collagen in the absence of the α2 integrin^28^. According to the confocal microscopy images, variant integrin α2E336A, unlike α2, did not participate in the formation of focal adhesion sites, but was mainly located in other plasma membrane areas (Fig. 3b) in a very similar manner as was seen in the cells plated on EV1 (Fig. 3a). Based on the quantitative image analyses performed with the BioimageXD software^29^, we detected the colocalization of wild type α2β1 with talin-1, α-actinin and vinculin, whereas α2E336A colocalized with talin-1 and α-actinin, but clearly less with vinculin (Fig. 3b,c). Furthermore, α2E336A did not localize to vinculin positive focal adhesions (Fig. 3c). Thus, there seems to be spatial association between the nonactivated, presumably bent α2β1 integrin and talin-1 although we have no evidence supporting direct protein–protein interaction. Importantly, the data support the idea that the adhesion sites formed by the interaction between the nonactivated integrin and EV1 are distinct compared to conventional focal adhesions. In the atypical adhesion sites, talin-1 does not sense the physical forces as it should as a link protein between integrins and actin microfilaments.

Talin may also be a critical factor in the activation of FAK. It is supposed to recruit FAK to the focal adhesions^9, 14^ together with paxillin^10^ and membrane-bound PIP_2_^11^. In the next series of experiments we created a lentivirus harbouring a talin-1 shRNA construct and used it to silence talin-1 expression in SaOS^α^ ^2+^ cells (Fig. 3d). In three independent experiments, the absence of talin-1 did not affect the activation of FAK mediated by the α2β1–EV1 interaction (Fig. 3d), indicating that FAK activation by nonactivated integrins is not talin-1 dependent. A previous report proposes that at least in nascent adhesions talin is not required in the initial phase of FAK phosphorylation^30^.

We have previously shown that EV1 interaction with the α2 integrin activates PKC-α signaling in host cells, and that the activity of both PKC-α and Rac1 are required for the uptake of EV1‒integrin complex in a micropinocytosis-like mechanism^15, 31, 32^. Here, we repeated these experiments and showed that the chemical inhibitors for PKC (Safingol) and Rac1 (NSC 23766) prevented EV1 cell entry (Fig. S5a,b). Previously, PKC-α has been indicated to activate FAK^33^. To test these pathways in the regulation of FAK, we treated cells with the Rac1 and PKC-α inhibitors prior to the plating of the cells on an EV1 coated surface. Safingol reduced FAK activation in four independent experiments (p = 0.03, Fig. 3e). The inhibition of PKC-α has been reported to prevent cell spreading, however, in our experiments the 30 min treatment did not yet reduce cell spreading remarkably (Fig. S5c). The Rac1 inhibitor neither reduced cell spreading on EV1 (Fig. S5c), nor had any effect on the activation of FAK (p = 0.48, Fig. 3f), suggesting that Rac1 is downstream of FAK also when FAK signaling is initiated by the nonactivated integrins.

### The adhesion mediated by the nonactivated integrins leads to a transient paxillin phosphorylation

**Figure 4:**
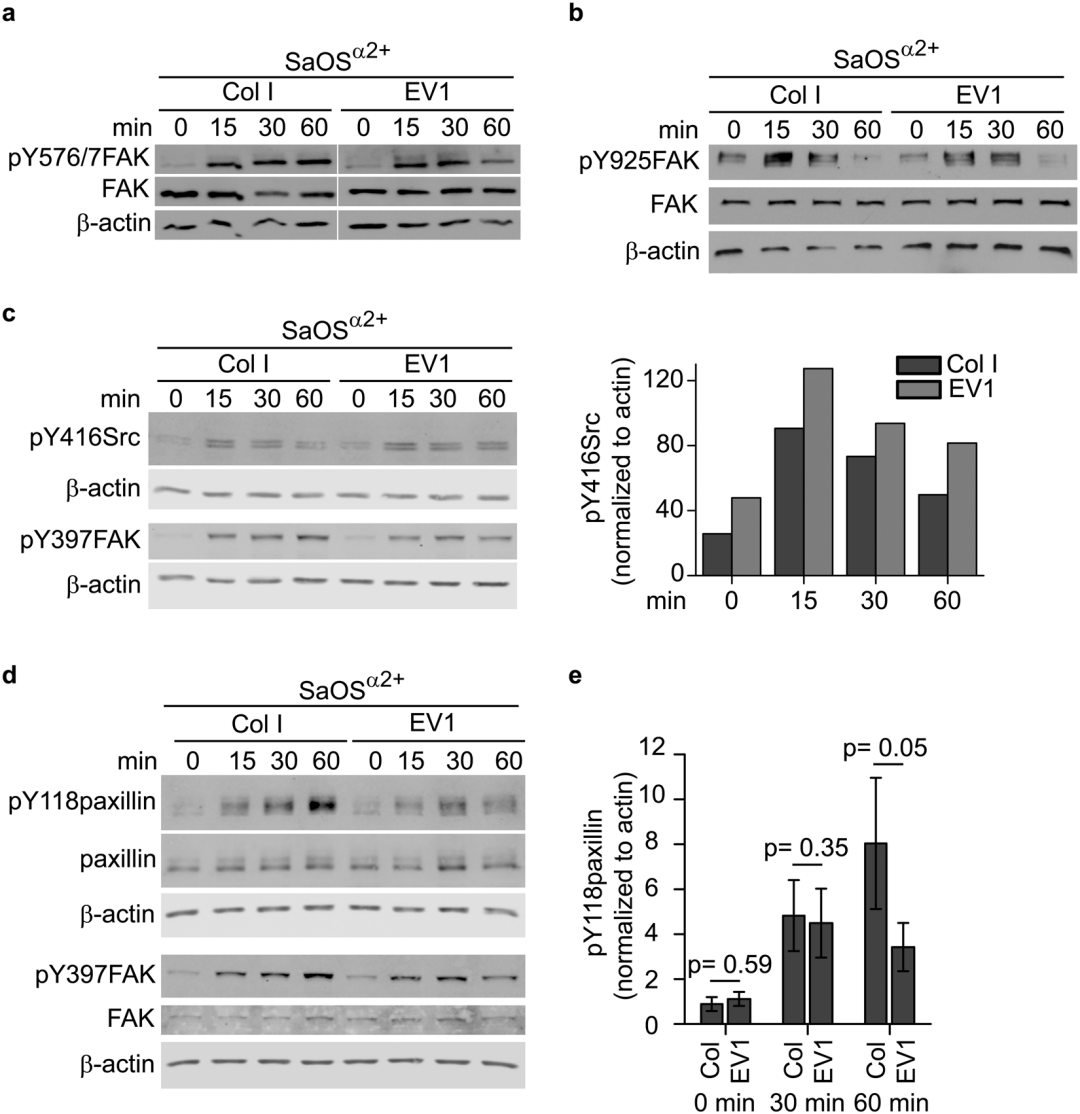
Nonactivated integrins induce full FAK activity. Western blotting of the phosphorylation of tyrosines 576/577 (**a**) and 925 (**b**) in FAK, tyrosine 416 in Src (**c**), and tyrosine 118 in paxillin (**d**) in SaOS^α^ ^2+^ cells plated on collagen I and on EV1. (**e**) Paxillin phosphorylation normalized to actin from three independent experiments described in (**d**). Figure shows mean values of activation and standard deviation at 0, 30 and 60 min time points.

FAK autophosphorylation at Y397 allows Src first to bind to FAK and then to phosphorylate the residues Y576 and Y577 in FAK. Fully activated FAK can then bind and phosphorylate other focal adhesion proteins^14^. In our model system we could detect that the interaction between α2 integrin and EV1 also induces the phosphorylation of Y567 and Y577 in FAK (Fig. 4a). We could also detect the phosphorylation of FAK Y925 and Src Y416 (Fig. 4b,c). Paxillin is one of the most important proteins interacting with activated FAK^34^, and indeed, we observed the phosphorylation of Y118 in paxillin when SaOS^α^ ^2+^ cells were plated on an EV1 coated surface (Fig. 4d). On collagen I, the phosphorylation of paxillin increased up to 60 min, whereas on EV1 the activation started to decline after 30 min (p = 0.05, difference between EV1 and COL I at 60 min time point, Fig. 4e). Thus, the nonactivated integrins can induce the full activation of FAK, but the downstream signaling events are transient by nature.

## Discussion

In the nonactivated conformation, integrin legs are together and the head piece of the receptor is facing the plasma membrane due to the bending of the legs at the “knee” sites. The bent integrin conformation was first described with β3 and β2 integrins and recently also β1 integrins have been supposed to take a similar state before the inside-out activation^35^. Previous papers have suggested that the nonactivated integrin conformations are not inactive, but can participate in biologically important functions. Integrins with the closed αI domains may mediate leukocyte rolling^36^ and, furthermore, bent collagen receptors on platelets bind to collagen I under shear stress conditions^4^. Recently, integrins in the bent conformation have also been linked to the regulation of fibronectin fibrillogenesis^2, 3^. The ligand interactions by the nonactivated integrins can be structurally explained by the flexible nature of the receptors. This is most evident in the nine out of 18 human integrin α subunits which recognize their ligands with an αI domain. According to the atomic structure of αXβ2 integrin, the αI domain seems to be quite flexibly bound to the head piece of the receptor and it may therefore reach ligands, even if the rest of the integrin is in a bent conformation^37^. Here, this study focuses on the α2β1, a collagen receptor, that also has an αI domain. Based on the *in vitro* experiments utilizing recombinant collagen-recognizing αI domains, the proteins in the closed, nonactivated conformation can bind to their ligands with a relatively high avidity^38–42^, again suggesting that the nonactivated integrin conformation does not prevent the ligand recognition.

The closed conformation integrin α2I domain can bind to EV1 with higher avidity than the activated, open conformation α2I domain^15^. Similarly, α2 integrin harbouring a loss-of-function E336A mutation is a better receptor for EV1 than the α2 integrin that contains a gain-of-function E318W mutation^15^. Therefore, we have suggested that α2β1–EV1 interaction model can be used to study the biology of nonactivated integrins in general. In this paper we report, that nonactivated α2β1 integrin can initiate a process leading to the autophosphorylation of Y397 in FAK. Four lines of evidence presented in this paper support the idea that no preactivation of integrins was needed: Firstly, FAK was phosphorylated after integrin binding to EV1, a ligand that we have previously shown to favour the nonactivated integrin conformation and not to induce the conformational activation of the receptors^15^. Secondly, a loss-of-function mutation E336A in the α2 integrin subunit did not prevent FAK activation. This is the mutation that is considered to keep the integrin in nonactivated conformation^19, 20^. Thirdly, EDTA did not prevent FAK activation, despite the fact that the absence of divalent cations has been reported to force the integrin to take the bent conformation^22^. Fourthly, here FAK phosphorylation took place in the absence of talin, a protein that is usually required for the integrin inside-out activation^26^.

EV1 has 60 potential α2I domain binding sites of which up to 10 sites can be simultaneously occupied, and therefore EV1 is capable of receptor clustering^15, 43^. Our previous reports have also speculated that clustering of the nonactivated α2β1 may alone be sufficient to activate signaling proteins such as PKC-α^32^. Accordingly, clustering of FAK proteins in a test tube and in cellular models leads to the autophosphorylation of Y397^10^. In addition, FAK can be activated by clustering antibodies for β1 integrins^44^. However, EV1 induced, nonactivated α2β1 integrin mediated FAK activation cannot be directly compared to the previously reported activation of FAK by clustering antibodies, since unlike EV1^15^ the antibodies may also cause a conformational activation of β1 integrins^44^.

In several cancers both FAK^45^ and α2β1 integrin^46^ transcription are enhanced. Elevated FAK phosphorylation has been reported to protect cancer cells from anoikis^47^ whereas increased α2β1 integrin expression has been found in cancer stem cells^48, 49^. Loss of FAK has also been shown to reduce cancer stem cell population in breast cancer^50^. Both FAK and α2β1 integrin overexpression promote cell migration, invasiveness and EMT^45, 46^. FAK can be activated in cancer cells through increased intracellular pH that leads to deprotonation of histidine residue 58, enhanced growth factor receptor signaling, increased matrix stiffness, and due to spontaneous dimerization and autophosphorylation^45^. Integrin clustering is, however, the most common mechanism to activate FAK. Thus, it is possible to speculate that ligand independent clustering of highly expressed integrins may lead to the activation of FAK in cancer cells. Our observations support such hypothesis, since we show that integrins can activate FAK through integrin clustering and without conformational activation.

Importantly, FAK activation after EV1–α2β1 interaction differs from the conventional activation mechanism seen after integrin binding to the ECM molecules. Firstly, the cell adhesion site seems to be different in its appearance in confocal microscopy and also in its molecular composition. The loose structures at the tip of cellular protrusions mediating the attachment to EV1 contained talin-1 and α-actinin, but not vinculin. The lack of vinculin in the adhesions and the lack of manifest microfilaments in the adherent cells suggest that talin is not bridging integrins to polymerized actin in a similar manner as in the conventional focal adhesions. Interestingly, one previous report indicates that the mechanism of FAK activation induced by the interaction between α2β1 integrin and collagen may have fundamental differences when compared to FAK phosphorylation initiated by the interaction between fibronectin and its receptor, since the first one is independent of mechanical tension but the latter is not^12^.

Here, FAK activation was talin-1 -independent, but required the activity of PKC. The mechanism of FAK activation during the formation of conventional focal adhesions is still unclear. The presently predominating working model stresses the role of talin^9, 14^. Here, the observation that talin-1 did not participate in FAK activation was not surprising given the fact that the role of talin in the formation of the adhesion site was clearly different when compared to the canonical focal adhesions. Furthermore, even in nascent adhesions FAK can be recruited to integrin adhesions without talin^30^. The inhibition of FAK phosphorylation by PKC inhibitors has been shown earlier^33, 51, 52^. However, their effect may in many model systems be indirect and due to e.g. reduced cell spreading. Interestingly, both PKC-α^53^ and FAK^25, 54^ can also directly bind to β1 integrin cytoplasmic tail. Therefore, integrin clustering may bring the two kinases in close contact with each other.

Another difference between FAK activation by the nonactivated and conventionally activated α2 integrins was the more transient nature of the first one. We repeatedly observed that FAK phosphorylation started to decline after 30 min, especially in the experiments with E366A variant of α2 integrin. The difference in paxillin phosphorylation at 60 min time point was even more significant.

To conclude, previous studies have shown that integrins can interact with ligands also without integrin preactivation. Here we report that such process can also initiate the phosphorylation of FAK. However, there are significant differences when compared to customary FAK activation. After binding to EV1, nonactivated α2β1 integrins form an atypical adhesion site in which FAK activation takes place via talin-1 -independent mechanism that requires PKC activity. Consequent signaling, namely paxillin phosphorylation, is more transient in nature than after the formation of conventional focal adhesions.

## Materials and Methods

### Cell culture

SaOS-2 cells were obtained from ATCC. HeLa OHIO cells, originally from ATCC, were a gift from Dr. Petri Susi from department of Virology, University of Turku, Finland. Cells were cultured in Dulbecco’s modified Eagle’s medium (DMEM) supplemented with 10% FCS, 2 mM Ultraglutamine 1, 100 U/ml penicillin and streptomycin. SaOS cells were stably transfected with wild type human integrin α2 and loss-of-function α2 (α2E336A) as described previously^15, 21, 24^. SaOS cells were stably transfected with α2/α1-tail that has been described earlier^24^. Transfected cells were grown under geneticin (250 μg/ml) selection.

### Echovirus 1

EV1 (Farouk strain, ATCC) was produced at the Department of Virology, University of Turku, Finland. EV1 was propagated in LLC cells and purified with sucrose gradient according to Abraham and Colonno^55^. To remove possibly remaining sucrose, virus patch was further purified with dialysis against PBS supplemented with 0.5 mM MgCl_2_ using Float-A-lyzer (MWCO 50 kDa, Spectrum Laboratories).

### shRNA

Talin1 (sh1:TRCN0000299020, sh2:TRCN0000299022) and FAK (TRCN000001617) were silenced with lentiviral based RNA interference (Mission shRNA, Sigma). Lentivirus particles were produced using third generation production system. pLKO1 plasmid containing the target sequence, together with packaging vectors (pMDL, pREV, pVSVG; Center of Biotechnology, Turku, Finland) were transfected into HEK-293T cells with Fugene 6 (Promega). Viral particle containing media was collected and stored in −20 °C. Target SaOS cells were infected with 1:1 ratio of fresh DMEM and virus media supplemented with 8 μg/ml polybrene. Media was changed the next day and 5 μg/ml puromycin was added the following day to enrich infected cells. Cells were used for experiment 96 h after infection.

### FAK, Src and paxillin activation on EV1 and collagen I surface

2.5 μg/cm^2^ EV1, 5 μg/cm^2^ Pure Col type I bovine collagen (Advanced BioMatrix), or 0.1 mg/ml poly-L-lysine in PBS was incubated on cell culture plate overnight at +4 °C, and plates were blocked with 0.1% BSA in PBS for 1 hour at +37 °C. Cells were grown in low serum (0.5%) overnight, and where indicated, treated with inhibitors, shRNA, or EDTA. Inhibitors for PKC (safingol, 10 µM, 30 min, Calbiochem) and Rac1 (NSC23766, 100 µM, 1 h, Santa Cruz) were added prior cell plating and kept during cell adhesion. 5 mM EDTA was added 10 minutes before plating the cells and kept during cell adhesion. When detaching the cells for the experiment, trypsin-EDTA was allowed to completely detach the cells. Trypsin was inhibited with equal volume of 1 mg/ml trypsin inhibitor, and cells were pelleted and washed, and then re-suspended in serum free DMEM. Cells were allowed to attach the virus coated surface for indicated time (15–60 min) and lysed for Western blot analysis. Control samples for Western blot (0 min) were pelleted and lysed without cell plating.

### Analysis of FAK, Src and paxillin activation

Cells were lysed in cold lysis buffer (10 mM Tris-HCl pH 7.4; 1% Triton X-100) including protease inhibitors (1 mM EDTA, 10 mM NaF, 10 μg/ml Aprotin, 10 μg/ml Leupeptin, 1 mM Na3VO4, 2 mM PMSF, 10 mM Na_4_P_2_O_7_) and phosphatase inhibitor tablet (Roche or Thermo Scientific). Lysates were centrifugated at 13000 G for 10 minutes at +4 °C and protein concentrations were measured using Bradford reagent (Bio-Rad Laboratories, Inc.). 10 to 30 μg of protein in Laemmli SDS-PAGE sample buffer was loaded on gels and proteins were separated with SDS-PAGE electrophoresis. Proteins were electrotransferred to nitrocellulose membrane and blocked with blocking buffer (5% milk powder, 1% BSA, TBST) for 1 hour. Phospho-FAK primary antibodies (FAK-phospho-Y397, FAK-phospho-Y576/Y577 and FAK-phospho-Y925, Cell Signaling), phospho-Src (Src-phospho-Y416, Cell Signaling) and phospho-paxillin antibody (paxillin-phospho-Y118) were incubated overnight at +4 °C in 5% BSA in TBST, and secondary antibodies (Horseradish peroxidase conjugated secondary antibodies, Santa-Cruz Biotechnology, Inc.; or Li-COR IRDye 680RD/800CW, Li-COR Biosciences) for 1 hour at room temperature in blocking buffer or TBST, respectively. Phospho-antibodies were removed with stripping buffer (Glycine-HCl, 2 times 10 min). Anti-FAK (BD Biosciences), anti-paxillin (BD Biosciences) and anti-actin (Santa-Cruz Biotechnology, Inc.) were incubated for 1 hour in RT. In shRNA silenced cells, also the levels of Talin 1 (Chemicon) were detected. SuperSignal West Pico and Femto Chemiluminescent substrate from Thermo Scientific were used to detect the bands with X-Ray-films, and Odyssey CLx from Li-COR Biosciences to detect fluorescent secondary antibodies. Band intensities were analysed with ImageJ or directly with Odyssey CLx, and phosphorylated FAK/paxillin was proportioned to either actin or total FAK/paxillin signal.

### Live imaging

24-well plates were coated with EV1 and type I collagen as described above, and blocked with 0.1% BSA. Cells were suspended in serum free DMEM and plated on wells. Images were immediately obtained with Zeiss Axiovert 200 M.

### Confocal microscopy

For confocal microscopy of cell adhesions, EV1 and type I collagen were coated either on 6 cm cell culture plates inside paraffin circle, or on plastic coated glass surface. Cells were allowed to attach for 1 hour and then fixed in 4% paraformaldehyde for 20 minutes at room temperature, and permeabilized with 0.2% Triton x-100 in PBS for 5 minutes. Cells attached on cell culture plates were immunolabelled in 50 μl drop inside paraffin circle with α2 integrin (rat, clone 430903, R&D Systems), β1 integrin (clone K20, sc-18887, Santa-Cruz Biotechnology, Inc.), talin-1 (MAB1676, Chemicon), and vinculin (mouse, Sigma) antibodies where indicated. Alexa-488 or 555 labelled secondary antibodies against mouse and rat (Invitrogen), were used. Actin stress fibers were visualized with Alexa-633 conjugated phalloidin (Invitrogen). Mowiol, including DABCO (Sigma), was added on top of the cells and high quality 13 mm cover glass was placed inside a circle on a cell culture plate. For imaging, the plate was inverted, attached on objective glass from the bottom, and imaged with Zeiss LSM 510 confocal microscope. Shown confocal microscopy images are not adjusted.

### Image analysis

To analyse the colocalization of α2-integrins with talin-1, α-actinin and vinculin, 10 to 20 SaOS^α^ ^2+^ and SaOS^α^ ^2E336A^ cells from three independent experiments were imaged. Image analysis was performed with BioImaxeXD batch processor function^29^. α2-integrin positive areas were defined with threshold function, and used as a mask to measure signal intensity of talin-1, α-actinin and vinculin channels in those areas. Talin-1, α-actinin and vinculin intensities in α2-positive area were divided by the signal intensity of the protein of interest in the total cell area (defined by hand using ROI function).

### Statistical analysis

Statistical analysis was performed using IBM SPSS Statistics software version 21. Paired Student’s t-test was used to determine the statistical significance of the results when two Western blot timepoints or two treatments in confocal microscopy were compared. When several Western blot timepoints were compared, the data was analysed with nonparametric Mann-Whitney *U* test and Bonferroni correction was used to correct false discovery rate.

## Electronic supplementary material

Supplementary info
