# Supplementary material for: Integrin α2β1 in nonactivated conformation can induce focal adhesion kinase signaling
Source: Sci Rep. 2017 Jun 13;7:3414. doi: 10.1038/s41598-017-03640-w (PMC5469853; doi:10.1038/s41598-017-03640-w)
Supplement: Supplementary file 1 — Supplementary info [file 41598_2017_3640_MOESM1_ESM.pdf]

SUPPLEMENTARY DATA

Integrin α2β1 in nonactivated conformation can induce focal adhesion kinase signaling

Maria Salmela[1], Johanna Jokinen[1,2], Silja Tiitta[1], Pekka Rappu[1], Holland Cheng[2] & Jyrki Heino[1,*]

[1]Department of Biochemistry, University of Turku, Turku, Finland
[2]Department of Molecular and Cellular Biology, University of California, Davis, CA, USA

*Corresponding author. Tel: +358 2 3336879; E-mail jyrki.heino@utu.fi

SUPPLEMENTARY METHODS

Covalent and noncovalent linking of EV1

Covalent linking of EV1 to cover glasses was performed at the University of Tampere in collaboration with Vesa Hytönen, according to their modified protocol for thin gels originally described in Buxboim *et al.*, 2010. Cover glasses were soaked in 0.1 M NaOH and aminosilanized with (3-aminopropyl)-triethoxysilane under vacuum. Next, the glasses were treated with Sulfo-SANPAH (1 mg/ml in Hepes) and glass surface was photoactivated with UV-light. After washing with Hepes (50mM, pH 8.5), EV1 in PBS (2.5 µg/cm2) was added on top of the cover glass and incubated at +4 overnight. For noncovalent linking, glasses were treated with 0.1 M NaOH.

Flow cytometry

SaOS cells were trypsinised, washed and suspended in blocking buffer (1 % FCS in PBS). Cells were incubated for 1 h with α2 integrin primary antibody (12F1, BD Biosciences) and for 1 h with FITC-conjugated anti-mouse secondary antibody (A16167, Novex, Life Tecnologies). As controls, FITC-labelled cells were used. Cells were washed and suspended in PBS, and α2 integrin levels were measured with FACS Calibur (BD Biosciences). Graphs were drawn with Flowing Software 2 (Turku Centre for Biotechnology, Turku, Finland).

Immunoprecipitation of α2-β1 heterodimer

$SaOS^{α2-}$, $SaOS^{α2+}$ and $SaOS^{α2E336A}$ cell monolayers were washed with ice cold buffer containing 150 mM NaCl, 1mM $CaCl_2$, 1mM $MgCl_2$, and Tris-HCl (pH 7.4). Cells from three 10 cm plates were collected with 1 ml of a buffer by scraping, and then pooled. Cells were pelleted by centrifugation (500 G, 4 min) and the pellets were solubilized with 0.5 ml of the same buffer containing 100 mM n-octyl-6-D-glycopyranoside (Sigma) on ice for 15 min with occasional vortexing. The lysates were cleared first by centrifugation (13000 G, 4 min, +4) and then by 1 hour incubation with G-Sepharose 4 Fast Flow beads (GE Healthcare) in the presence of bovine serum albumin (0.5 mg/ml) and Triton x-100 (0.5 v/v%). The resulting supernatants were immunoprecipitated with α2 integrin 12F1 antibody (mouse anti-human, BD Biosciences) for 12 hours and the complexes were recovered by binding to G-Sepharose beads for 1 hour. The beads were washed three times with a buffer containing 150 mM NaCl, 25 mM Tris-HCl (pH 7.4), 0.1 v/v% Triton x-100 and 1 mg/ml bovine serum albumin, and twice with a buffer containing 150 mM NaCl and 25 mM Tris-HCl (pH 7.4). The immunoprecipitates were detached from the beads with a non-reducing Laemmli-buffer (with SDS) by boiling for 5 min and analysed by electrophoresis on SDS-PAGE gels. Proteins were electrotransferred to nitrocellulose membrane and the membrane was blocked with blocking buffer (5 % milk powder, 1 % BSA, TBST) for 1 hour. β1 integrin was detected with rabbit β1 antibody R332 (Heino *et al.*, 1989) in blocking buffer for 1 hour in RT and using a donkey anti-rabbit secondary antibody (Li-COR IRDye 800CW, Li-COR Biosciences). Antibody was removed with stripping buffer (Glycine-HCl, 2 times 10 min). α2 integrin was detected with rat anti-human antibody (R&D Systems, clone 430903) in a blocking buffer for 1 hour in RT and using goat anti-rat secondary antibody (Li-COR IRDye 680RD, Li-COR Biosciences). Odyssey CLx from Li-COR Biosciences was used for the detection of fluorescent secondary antibodies.

Inhibitor-effect on EV1 infection

Cells were treated with Safingol (10 µM, 30 minutes, Calbiochem) and NSC 23766 (100 µM; 1 hour; Santa Cruz Biochemistry, Inc) prior to and during EV1 infection. Inhibitors were added to the cells in DMEM without serum and incubated at +37 °C. For EV1 infection assay adherent $SaOS^{α2+}$ cells were incubated with EV1 in serum free DMEM on ice for 1 hour. Unbound virus was removed by washing the cells with PBS, and DMEM including 10 % FCS was added. Virus infection was allowed to proceed for 5 to 6 hours at +37 C. Cells were fixed in 4 % paraformaldehyde (Thermo Scientific) for

20 minutes at room temperature, permeabilised with 0,2 % Triton x-100 in PBS for 5 minutes, and labelled with anti-EV1 antibody [17] and DRAQ5 (Cell Signaling) or DABI nuclear label. Cells were imaged with Leica TCS SP5 Matrix or Zeiss Axiovert 200M. Number of infected cells was analysed with BioImage XD [29].

Analysis of EV1 infected cells

For the analysis of EV1 infection after PKCα and Rac1 inhibition, three replicate wells and 9 images per well were analysed in each experiment. Image analysis was performed with BioImage XD Batch processor –function [29]. Percent of infected cells was calculated as a ratio of EV1 positive areas versus DAPI/DRAQ5 labelled nuclei. Noise from both channels was reduced using Gaussian smoothing – filter. Thresholds for EV1 and DAPI/DRAQ5 channels were determined manually. EV1 channel was segmented using Connected component labelling, and DAPI/DRAQ5 channel using Object separation. Infection percent was calculated using the value Number of segmented objects from each image.

SUPPLEMENTARY REFERENCES

Buxboim A, Rajaqopal K, Brown AEX, Discher DE (2010) How deeply cells feel: methods for thin gels. *J Phys Condens Matter* **22**: 1–19.

Heino J, Ignotz RA, Hemler ME, Crouse C, Massagué J (1989) Regulation of cell adhesion receptors by transforming growth factor-beta. Concomitant regulation of integrins that share a common beta 1 subunit. *J Biol Chem* **264**: 380–388.

SUPPLEMENTARY FIGURES

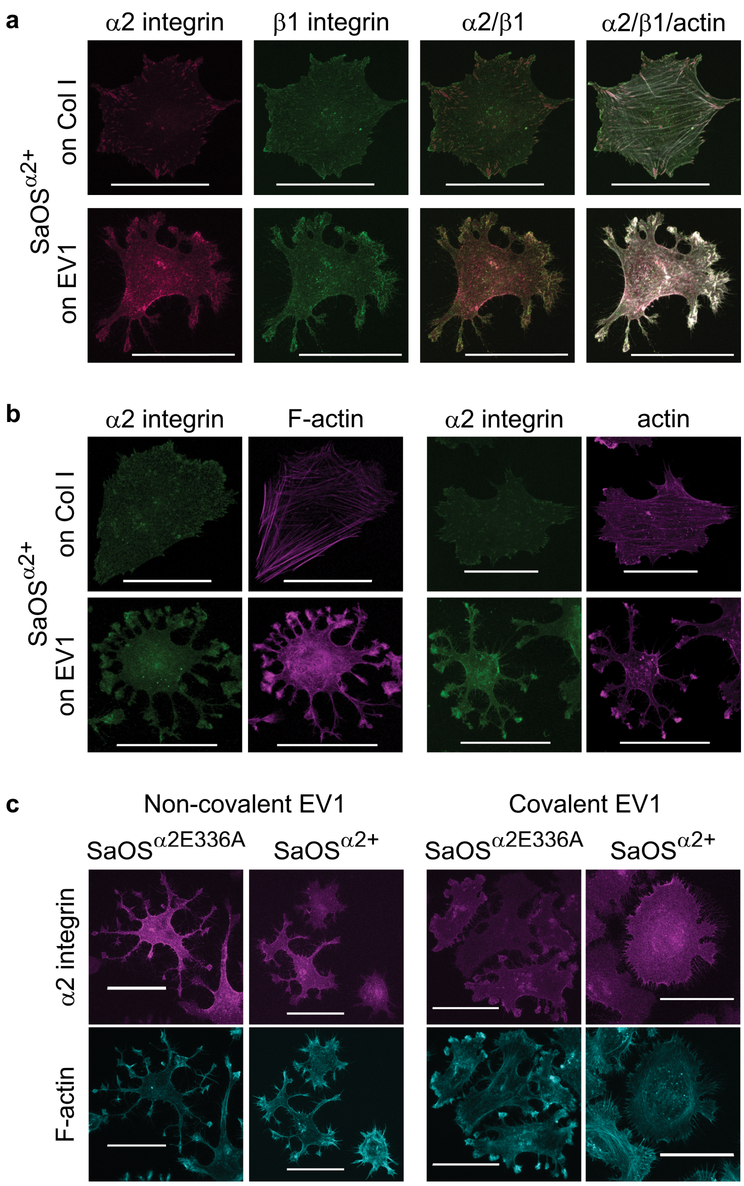

Figure S1 - SaOS cells do not form focal adhesions or stress fibers on EV1 coated surface. (**a**) Confocal microscopy images of SaOS$^{\alpha2+}$ cells plated on Collagen I and EV1 coated surface (60 min) α2 integrins, β1 integrins and actin stress fibers were labelled using specific antibodies and phalloidin, respectively. Scale bar 50 µm (**b**) Confocal microscopy images of SaOS$^{\alpha2+}$ cells plated on Collagen I and EV1 coated surface (60 min). α2 integrins, globular actin and actin stress fibers were labelled using specific antibodies and phalloidin, respectively. Scale bar 50 µm. (**c**) Confocal microscopy images of SaOS$^{\alpha2+}$ and SaOS$^{\alpha2E336A}$ cells plated on EV1 coated surface after either non-

covalently or covalently linking EV1 to glass. α2 integrins were labelled using specific antibody and actin stress fibers using Alexa-633 conjugated phalloidin. Scale bar 50 μm.

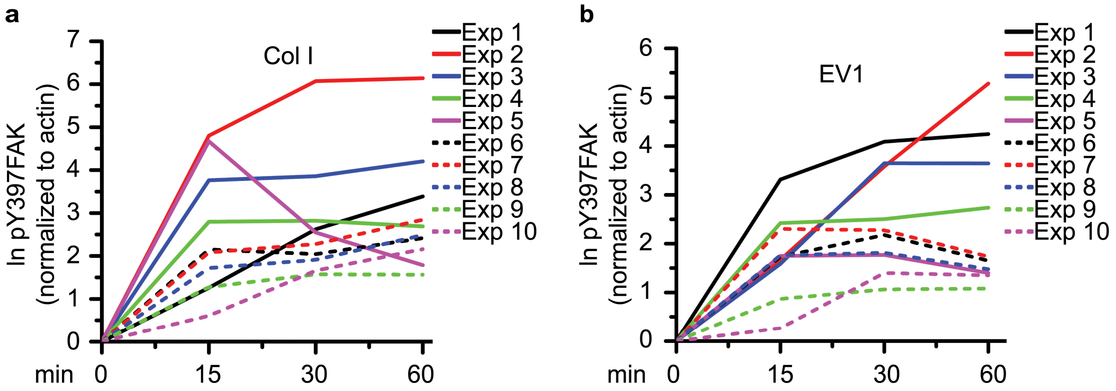

Figure S2 - FAK is phosphorylated both on Col and EV1 coated surfaces.

Figure shows ten independent Western blotting experiments of FAK Y397 phosphorylation in SaOS$^{\alpha 2+}$ cells spreading on an immobilized layer of EV1. Phospho-FAK levels were normalized first against actin and then against the smallest value in the experiment for the comparison between experiments.

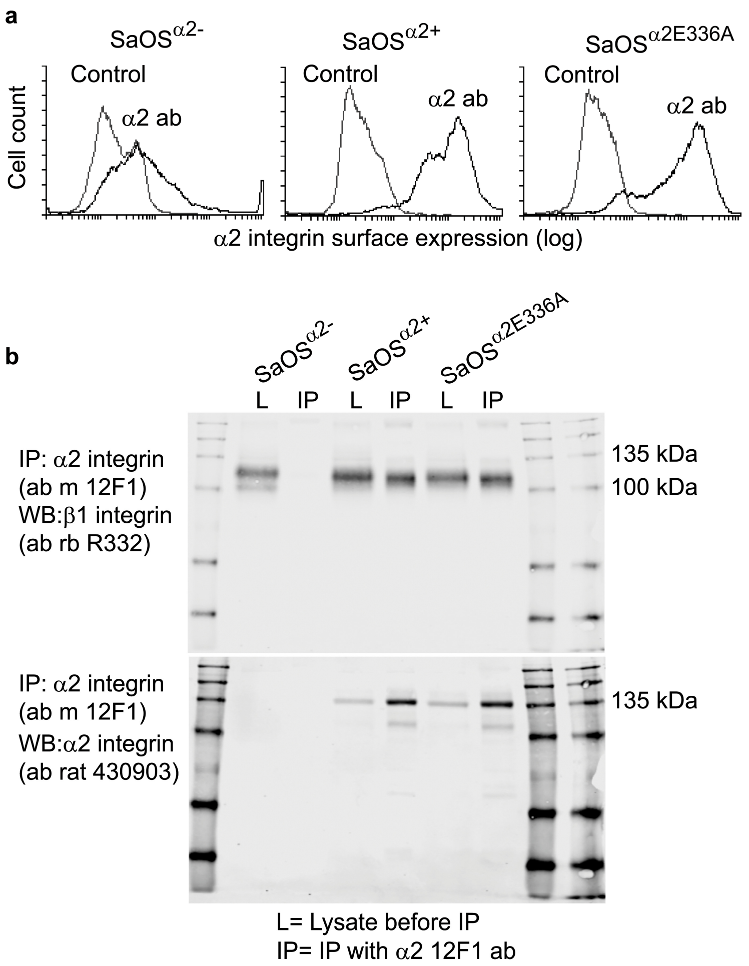

Figure S3 – Cell surface expression levels of transfected α2-integrins and α2-β1 dimerization. (**a**) Flow cytometric analysis of the surface expression of transfected α2-integrin in SaOS$^{α2+}$ and SaOS^{α2E336A} cells, as well as lack of α2 in SaOS$^{α2-}$ cells. Cell surface integrins were labelled with α2 specific antibody and FITC-conjugated secondary antibody, and cells were analysed with FACS Calibur. (**b**) Immunoprecipitation of α2 integrin from SaOS$^{α2-}$, SaOS$^{α2+}$ and SaOS^{α2E336A} cells and co-immunoprecipitation of β1 subunit. α2 was immunoprecipitated with 12F1 antibody, following western blotting of β1 subunit (rabbit R332) and α2 subunit using a different α2 recognizing antibody (R&D clone 430903).

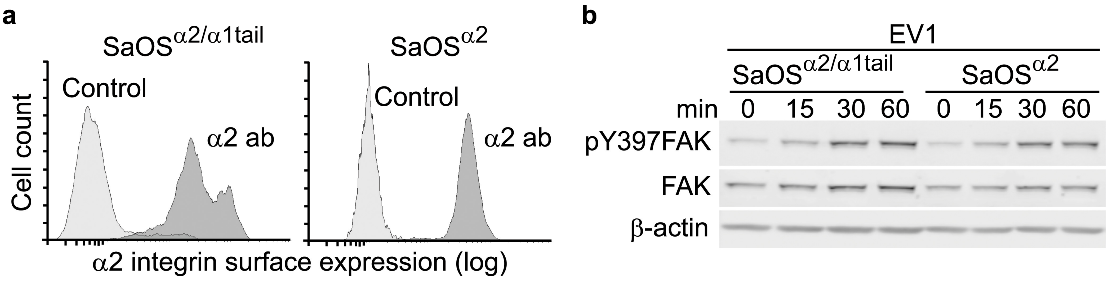

Figure S4 - FAK phosphorylation is not α-tail selective.

(**a**) Flow cytometric analysis of the surface expression of transfected α2-integrin in SaOS$^{\alpha2/\alpha1tail}$ and SaOS$^{\alpha2+}$ cells. Cell surface integrins were labeled with α2-specific antibody and FITC-labeled secondary antibody and analysed with FACS Calibur flow cytometer. (**b**) Western blotting analysis of FAK phosphorylation at Y397 in SaOS$^{\alpha2+}$ and SaOS$^{\alpha2/\alpha1tail}$ cells plated on EV1.

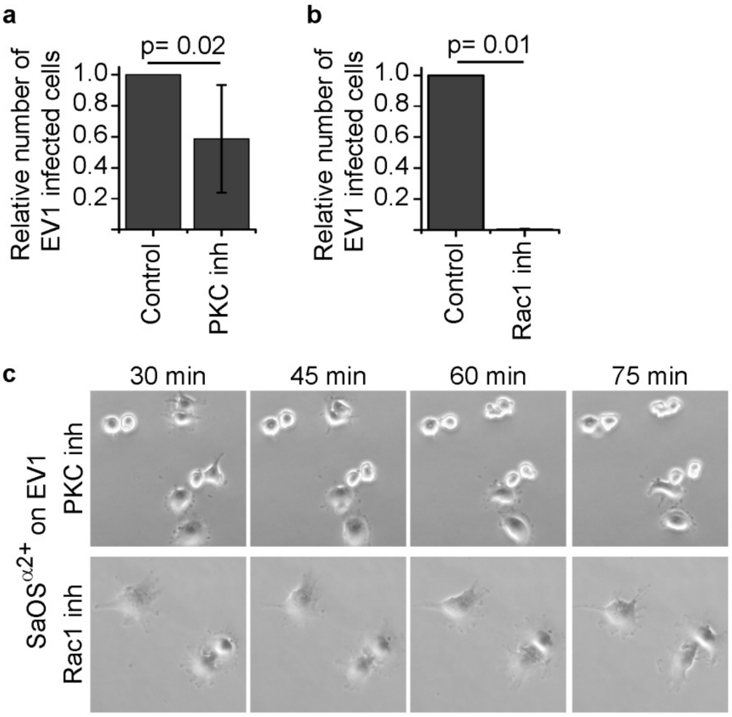

Figure S5 - Inhibition of PKC and Rac1 prevent EV1 entry but not cell adhesion to immobilized layer of EV1.

(**a**) Calculation of the relative number of EV1 coat antibody positive SaOS$^{\alpha2+}$ cells after incubating cells with EV1 particles for 6 h. In parallel experiments SaOS$^{\alpha2+}$ cells were treated with PKC inhibitor safingol (10 μM, 30 min), or with Rac inhibitor NSC23766 (100 μM, 1 h) prior to and during incubation with EV1. Results from five (PKC) or four (Rac1) independent experiments were analysed

with two-tailed paired Student's t-test and data are presented as mean value +/- SD. (**b**) Phase contrast microscope images of SaOS$^{\alpha2+}$ cells plated on an immobilized layer of EV1 after treating the cells with either PKC inhibitor (safingol, 10 μM, 30min), or Rac1 inhibitor (NSC23766, 100 μM, 1 h), prior to and during cell plating. Cells were photographed after 30, 45, 60, and 75 min.
